# Pharmaceutical companies and healthcare providers: Going beyond the gift – An explorative review

**DOI:** 10.1371/journal.pone.0191856

**Published:** 2018-02-07

**Authors:** Tom Latten, Daan Westra, Federica Angeli, Aggie Paulus, Marleen Struss, Dirk Ruwaard

**Affiliations:** 1 Department of Health Services Research, Care and Public Health Research Institute (CAPHRI), Faculty of Health, Medicine and Life Sciences, Maastricht University, Maastricht, The Netherlands; 2 Department of Organization Studies, School of Social and Behavioral Sciences, Tilburg University, Tilburg, The Netherlands; York University, CANADA

## Abstract

**Introduction:**

Interactions between pharmaceutical companies and healthcare providers are increasingly scrutinized by academics, professionals, media, and politicians. Most empirical studies and professional guidelines focus on unilateral donor-recipient types of interaction and overlook, or fail to distinguish between, more reciprocal types of interaction. However, the degree of goal alignment and potential for value creation differs in these two types of interactions. Failing to differentiate between these two forms of interaction between pharmaceutical companies and healthcare providers could thus lead to biased conclusions regarding their desirability. This study reviews the empirical literature regarding the effects of bilateral forms of interactions between pharmaceutical companies and healthcare providers in order to explore their effects.

**Material and methods:**

We searched two medical databases (i.e. PubMed and Cochrane Library) and one business database (i.e. EBSCO) for empirical, peer-reviewed articles concerning any type of bilateral interaction between pharmaceutical companies and healthcare providers. We included quantitative articles which were written in English and published between January 1^st^, 2000 and October 31^st^, 2016, and where the title or abstract included a combination of synonyms of the following keywords: pharmaceutical companies, healthcare providers, interaction, and effects.

**Results:**

Our search results yielded 10 studies which were included in our analysis. These studies focused on either research-oriented interaction or on education-oriented interaction. The included studies reported various outcomes of interaction such as prescribing behavior, ethical dilemmas, and research output. Regardless of the type of interaction, the studies either reported no significant effects or ambivalent outcomes such as affected clinical practice or ethical issues.

**Discussion and conclusion:**

The effects of bilateral interactions reported in the literature are similar to those reported in studies concerning unilateral interactions. The theoretical notion that bilateral interactions between pharmaceutical companies and healthcare providers have different effects given their increased level of goal alignment thus does not seem to hold. However, most of the empirical studies focus on intermediary, provider-level, outcomes such as altered prescribing behavior. Outcomes at the health system level such as overall costs and quality of care are overlooked. Further research is necessary in order to disentangle various forms of value created by different types of interactions between pharmaceutical companies and healthcare providers.

## Introduction

Cooperation between various organizations through inter-organizational relations has been described both as a hallmark and necessity in the healthcare industry [1, 2]. Although inter-organizational cooperation has been studied through various theoretical lenses [3], some of which appear more applicable to healthcare than others [4], scholars generally agree that organizations are embedded in various networks of inter-organizational relations [5, 6] and that these are crucial to organizational performance [7, 8]. A growing field of interest within the inter-organizational relations literature is that of cooperative relations between for profit and not-for-profit organizations, also referred to as cross-sectoral organizations or public-private partnerships [9, 10]. Scholars typically argue that for-profit and not-for-profit organizations collaborate in order to leverage complementary resources and institutional logics in order to create joint or social value and solve complex social problems [5, 11–13].

Within the healthcare context, interactions between pharmaceutical companies and healthcare providers (comprising both healthcare organizations as well as professionals) arguably constitute the most widely recognized form of relations between for-profit and not-for-profit organizations. These types of interactions occur on a large scale. Research from several countries has shown that more than eighty percent of physicians have interactions with pharmaceutical companies and one-fourth of US biomedical researchers receive industry funding for example [14–17]. Interactions between pharmaceutical companies and healthcare providers are increasingly scrutinized by academics, professionals, media, and politicians [18–23] and scholars have not reached a consensus about whether they indeed have beneficial effects and enhance social value [24, 25]. Following the social value-creation logic, various scholars argue that interaction between pharmaceutical companies and healthcare providers could foster the development of new medicines which will ultimately benefit patients [24, 26, 27]. Some empirical studies have furthermore shown that interaction between pharmaceutical companies and healthcare providers enhances technological innovation, fosters knowledge creation, aids disease control, and reduces polypharmacy issues [24, 28]. Conversely, others view the opposing logics in both organizations as insurmountable and suggest that interactions between pharmaceutical companies and healthcare providers are primarily geared towards promoting the products of the pharmaceutical company, which conflicts with the social responsibility of healthcare providers. Empirically, studies find that interaction between pharmaceutical companies and healthcare providers could result in a conflict of interests (COI), negatively alter physicians’ prescribing behavior, or result in a negative perception towards professionals [22, 29–35]. As a result, several guidelines have been created to help professionals navigate the opposing explanations of interactions with pharmaceutical companies [36–38].

Interactions between pharmaceutical companies and healthcare providers take various forms, ranging from personal gifts, free samples, visits by representatives, and marketing directed towards physicians to collaborative drug development, funding for continuing medical education (CME), sponsorships, and research-funding [20, 39]. Each of these interactions carries distinct features and characteristics. Within the inter-organizational relations literature, a common way to distinguish between different types of interactions between for-profit and not-for-profit organizations is through Austin and Seitanidi’s [10, 40] value creation spectrum and collaboration stages. The authors argue that collaboration between for-profit and not-for-profit organizations can result in associational value, transferred resource value, interaction value, and synergistic value. According to Austin and Seitanidi, the creation of each of these types of value is contingent on the type of interaction in which the for-profit and not-for-profit organization are engaged. In philanthropic collaboration, for-profit organizations predominantly transfer resources, typically in monetary terms, to the not-for-profit organization with limited to no exchange occurring between partners. In these collaborations transferred resource value and associational value do not enhance value-creating potential, and carry the least synergistic value [9]. In transactional collaboration, organizations’ interests are more closely linked, leading to bilateral exchanges of more specialized resources. In integrative collaborations, organizations exchange key assets in a conjoined fashion, generating greater synergistic value. Lastly, transformational collaborations constitute the most advanced stage of collaboration and are categorized by shared learning between the organizations, rather than mere resource exchanges. This type of collaboration is argued to have the strongest outcomes in terms of social value creation and bettering the lives of people.

In the context of interactions between pharmaceutical companies and healthcare providers, gifts (i.e. from the former to the latter) can best be described as philanthropic (i.e. donor-recipient) interactions. Although the motives of pharmaceutical companies might transcend, or even neglect, actual philanthropy [40], the resource flow in these types of interaction has a clear unilateral pattern. That is, the pharmaceutical company typically donates resources (often in the form of free merchandise) to healthcare providers without any resources being formally reciprocated. Conversely, interactions in the form of educational arrangements, licensing, or event sponsorships are best categorized as transactional interaction. In these types of interactions, healthcare providers are generally required to reciprocate resources in some form or the other (e.g. time). Lastly, joint research undertaken by pharmaceutical companies and healthcare providers falls within the realm of integrative transformational collaboration. That is, both need to devote considerable resources in a coordinated fashion to the project.

Despite the fact that various types of interaction between pharmaceutical companies and healthcare providers exist and that these occupy different collaborative stages, research [e.g. 20, 22] and practical guidelines [e.g. 36, 38] in this field rarely acknowledge these differences as such. However, failing to differentiate between unilateral (i.e. philanthropic) and bilateral (i.e. transactional, integrative, or transformational) types of interaction between pharmaceutical companies and healthcare providers provides an incomplete image of the effects of interactions between both industries. Consequently, interactions between pharmaceutical companies and healthcare providers are potentially over-generalized and over-scrutinized. The main aim of our study is thus to understand the effects of bilateral interactions between pharmaceutical companies and healthcare providers. We seek to contribute to the ongoing debate on this issue in two ways. Firstly, we make a clear distinction between the nature of interactions between pharmaceutical companies and healthcare providers. Consequently, the results of this study allow for a clearer comparison between the effects of bilateral, as opposed to unilateral, interactions between pharmaceutical companies and healthcare providers, as well as for a more nuanced understanding of the desirability of these interactions. Following the adoption of the distinction between unilateral and bilateral interactions, we secondly seek to integrate the knowledge produced in the business literature with that produced in the medical field. Combining both perspectives provides a more comprehensive understanding of the phenomenon.

## Materials and methods

### Search strategy

We studied the effect of bilateral interactions between pharmaceutical companies and healthcare providers by systematically reviewing the quantitative evidence regarding these interactions. Our review followed the PRISMA guidelines [41]. We searched two medical databases, namely PubMed and the Cochrane Library. In order to avoid retrieving a wide range of pharmacologic studies, which are not the focus of our study, we refrained from searching the Embase database. We did however search the business database EBSCO, which covers multiple databases such as Business Source Complete, MEDLINE, CINAHL, and Econlit. We deliberately refrained from using snowball sampling techniques to further our sample following the work of Horsly et al. [42], indicating that there is limited evidence to support this approach in reviews.

The PubMed, Cochrane Library and EBSCO databases were all searched for empirical, peer-reviewed articles which used a quantitative analysis, and were written in English. The title or abstract of articles had to include a combination of the following keywords: pharmaceutical companies, healthcare providers, interaction, and effects, or variations to these keywords (see Table 1 for a list of the specific keywords used). In the PubMed database, the relevant Medical Subject Heading (MeSH) terms were furthermore used (see Table 1 for a specification). Lastly, we included only those studies published between January 1^st^, 2000 and October 31^st^, 2016. Articles prior to 2000 were not included for two reasons. Firstly, Wazana [20] published an extensive review on various types of interaction between pharmaceutical companies and individual healthcare providers in 2000. Focusing on the post-2000 period hence avoids duplication of this work. Furthermore, the uptake in interest surrounding collaboration between for-profit and not-for-profit organizations has arguably been sparked by Austin’s work in 2000 [40] introducing the notion of various collaborative stages in these interactions. As such, we considered it likely that most of the studies in the business literature recognized this distinction from 2000 onwards.

**Table 1:** Search strategy PubMed (with MeSH), Cochrane Library and EBSCO.

Keywords	Synonyms
Pharmaceutical companies	pharmaceutical industr* OR pharmaceutical industry [MeSH] OR pharmaceutical industries [MeSH] OR drug indstr* OR drug industry [MeSH] OR drug industries [MeSH] OR drug compan* OR pharmaceutical compan*)
Healthcare providers	healthcare provider [MeSH] OR healthcare providers [MeSH] OR Health personnel [MeSH] OR physician OR physicians OR general practitioner [MeSH] OR general practitioners [MeSH] OR medical specialist* OR healthcare professional* OR health professional* OR doctor OR doctors OR medical doctor* OR healthcare organization* OR healthcare institution* OR hospital OR hospitals OR healthcare practice* OR healthcare industry [MeSH] OR healthcare industries [MeSH] OR general practice [MeSH]
Interaction	interaction* OR collaboration* OR cooperation* OR public private cooperation [MeSH] OR cooperative* OR collaborative* OR cooperative behavior [MeSH] OR cooperative behaviors [MeSH] OR cooperative behavior* OR cooperative behaviour* OR relation* OR partner* OR public private partnership [MeSH] OR payment* OR grant* OR grants [MeSH] OR sponsor* OR alliance* OR strategic alliance* OR funding* OR contact* OR association* OR connection* OR transaction* OR synerg* OR coalition*
Effect	effect* OR consequence* OR outcome* OR result* OR impact* OR influence* OR conclusion* OR implication*

### Study selection

Only those studies which met all of the inclusion criteria (see Table 2 for a specification) were considered relevant to our study. Most importantly, the studies had to focus on an interaction which was clearly identifiable as being bilateral in nature. Hence, studies had to contain a clear description of the interaction under investigation and only those studies which focused on interactions which involved mutual resource exchanges were considered relevant to our research question. Examples of such bilateral interactions include continuing medical education and joint research projects. In the former case, professionals have the opportunity to update their medical knowledge while the pharmaceutical company has access to the professional to market their products. In the latter case, the pharmaceutical company is provided with evidence of whether or not their product constitutes an advancement over existing treatments, while it allows professionals to enhance the quality of healthcare delivery if this is indeed the case, acquire representative insights in the product, and advance their academic career. Studies which focused on unilateral interactions, a one-directional transfer of (monetary) resources, were excluded from our analysis. Examples of such interactions include gifts to physicians and visits of sales (i.e. pharmaceutical) representatives (PSRs). Although visits of PSRs can be considered a relevant source of information to some physicians [43, 44], the main purpose of PSR visits is to provide physicians with samples or gifts [20] and physicians do not always consciously make the decision to be visited.

**Table 2:** Inclusion and exclusion criteria.

	Inclusion Criteria	Exclusion criteria
**Database**	- Search terms (See Table 1 for the search terms in PubMed and EBSCO) - Year of publication between January 2000 and October 31^st^, 2016 - Language: English - Peer-reviewed	N/A
**Title / Abstract**	The abstract or title of each article had to include a combination of the following keywords: pharmaceutical companies, healthcare providers, interaction, and effects, or variations to the preceding nouns.	- Explicitly mention only unilateral types of interaction or no clear distinction. - Explicitly mention a different aim then investigating an effect of interaction. - Non-empirical study. - Non- quantitative study. - Commentaries, reviews, opinion articles.
**Full-texts**	The article empirically investigates the effect of bilateral interaction between pharmaceutical companies and healthcare providers.	- Non-empirical studies. - Non-quantitative studies. - Studies on unilateral interaction or no clear distinction. - Other stakeholders. - Studies that only described the prevalence. - Studies that describes no interaction.

Secondly, articles were only included in our review in case the interaction occurred between pharmaceutical companies and healthcare providers (i.e. actively practicing healthcare professionals or healthcare organizations). Interactions between pharmaceutical companies and non-active professionals such as medical students were excluded.

Lastly, studies had to specifically address an identifiable outcome of bilateral interactions between pharmaceutical companies and healthcare providers in order to be eligible for inclusion in our review. Examples of such outcomes include the effect of bilateral interaction on physicians’ prescribing behavior or integrity as well as patients’ perception towards healthcare providers. Our study thus specifically focuses on which potential effects have been researched and which of these effects are supported by quantitative evidence. In case a study focused on unilateral as well as bilateral interaction, it is only eligible for inclusion when the effects of both types were clearly differentiated. For example, CME leads to effect X, educational training to effect Y, and visits of pharmaceutical representatives or gifts leads to effect Z. The articles were reviewed by two reviewers (T.L. and D.W.). In cases of disagreement the inclusion or exclusion of a particular study was discussed by both reviewers until a consensus was reached.

### Assessment of study quality

To assess the quality of the included empirical studies, each of the included studies was assigned a score based on the quality rating scheme of the Oxford Centre for Evidence-based Medicine for ratings of individual studies [45]. This rating scale is a proven, systemized approach which gives a score ranging from 1–5. In this rating scale, a score of 1 is an indication of high study quality (i.e. RCTs or meta-analyses), whereas a score of 5 indicates low quality (i.e. case reports). The scale was not used as a formal inclusion criteria, but served as a tool to get insight in the quality of the available literature regarding bilateral interaction between pharmaceutical companies and healthcare providers.

## Results

### Included studies and characteristics

**Fig 1:**
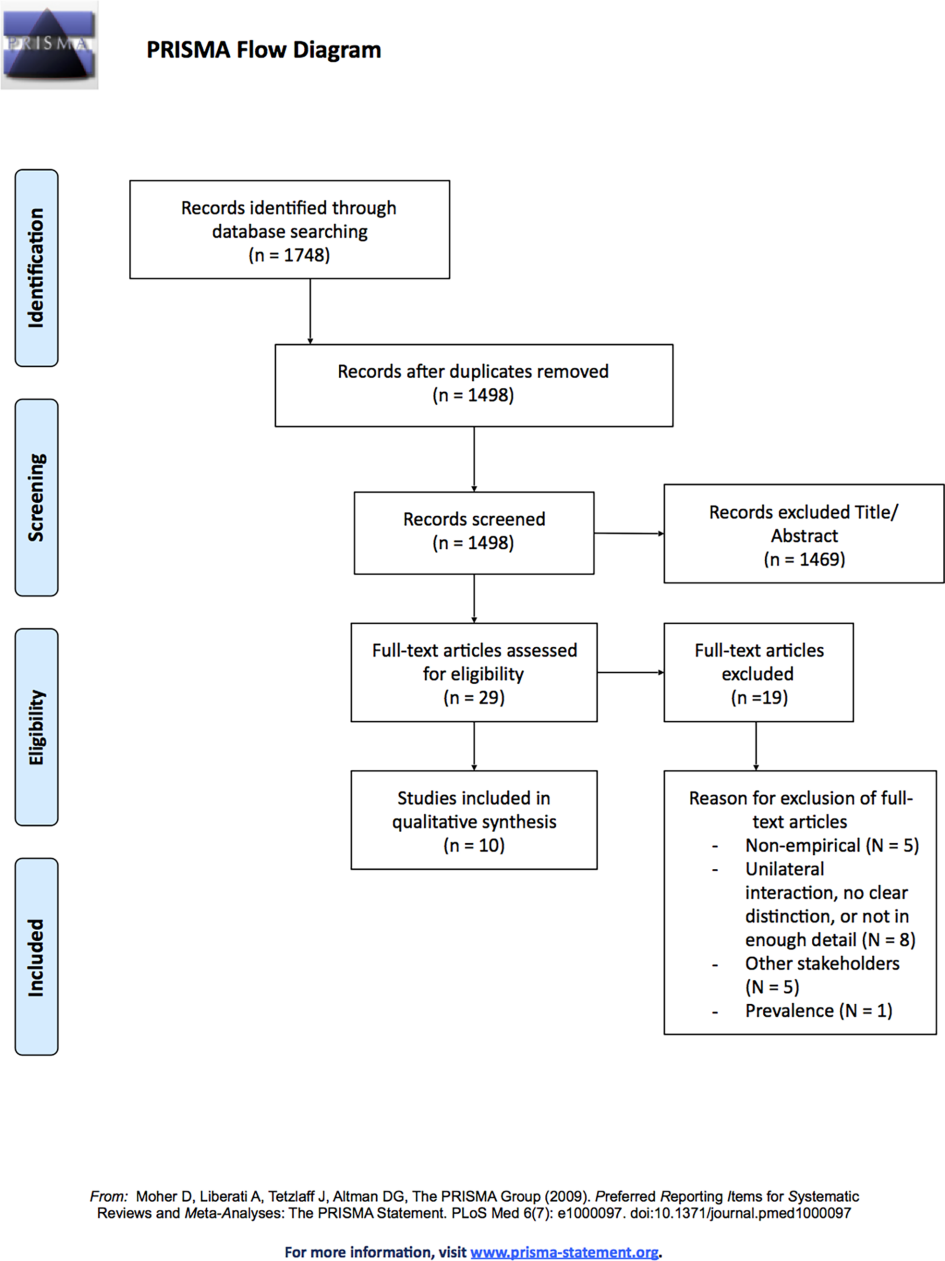
PRISMA flow diagram.

After elimination of duplicates, our search strategy identified a total of 1,498 studies. Initially, the eligibility of all articles was checked by reviewing the title and abstract of each study. This resulted in 29 studies, of which the full-text was assessed. Assessing the full text of these articles led to the exclusion of 19 additional articles. Exclusion of these additional articles was due to various reasons including: (A) the study was not empirical (N = 5), (B) the study only focused on unilateral interaction, did not made a clear distinction between unilateral and bilateral interaction, or the study did not describe the interaction between pharmaceutical companies and healthcare providers in enough detail (N = 8), (C) the study focused on other stakeholders such as healthcare authorities (N = 5), and (D) the study only focused on the prevalence of relations between pharmaceutical companies and healthcare providers but not on their effect (N = 1). As a result, a total of 10 empirical studies regarding the effects of bilateral interaction between pharmaceutical companies and healthcare providers were included in our review [18, 46–54]. The process of including and excluding articles is graphically represented by the PRISMA-diagram in Fig 1.

Most of the included studies in our review utilized a cross-sectional research design (N = 8). Additionally, one study had a quality rating of one [50], one study had a quality rating of three [46], seven studies had a quality rating of four [18, 47, 48, 51–54], and one study had a quality rating of five [49]. Table 3 provides an overview of each study’s characteristics.

**Table 3:** Characteristics of the selected studies.

Study, year	Site	Population (n)	Type of interaction	Type of effect	Study designs	Quality rating score
**Andersen, Kragstrup [46], 2006**	Denmark	Patients treated (5,439 case, 59,574 control)	Research-oriented (sponsoring)	Prescribing behavior	Retrospective cohort study	3
**Choudhry, Stelfox [47], 2002**	N-American and European societies	authors of Clinical Practice Guidelines (CPG) (100)	Research-oriented (CPGs)	Ethical dilemma	Cross-sectional survey	4
**Fisher and Kalbaugh [54], 2012**	United States	Informants (63)	Research-oriented (contract research)	Ethical dilemma	Cross-sectional qualitative study	4
**Glass [48], 2004**	United States	US physicians participating in phase 3 trial (2,108)	Research-oriented (clinical grant)	Prescribing behavior	Cross-sectional quantitative study	4
**Gray [49], 2013**	United States	University medical professor (1)	Research-oriented (funding)	Ethical dilemma	Case study (cross-sectional)	5
**Henry, Doran [53], 2005**	Australia	medical specialists (823)	Research-oriented (sponsoring)	Ethical dilemma	Cross-sectional quantitative study	4
**Myers, Shaheen [50], 2007**	Canada	physicians and nurses (229)	Research-oriented (sponsoring)	Research output	Cross-sectional randomized trial	1
**[51], 2016**	United States	Otolaryngologists (1,515)	Research-oriented (support)	Research output	Quantitative study	4
**Taylor, Huecker [18], 2016**	United States	US ophthalmologists (3011)	Education-oriented	Prescribing behavior	Cross-sectional quantitative study	4
**Yeh, Franklin [52], 2016**	United States	Massachusetts physicians (2444)	Education-oriented	Prescribing behavior	Cross-sectional quantitative study	4

### Types of interaction

The studies included in our review were categorized into two categories based on the type of interaction under study. These categories were; (a) education-oriented interaction [18, 52] (N = 2), and (b) research-oriented interaction [46–51, 53, 54] (N = 8). Interactions which were focused on increasing professionals’ (e.g. physician or nurse-practitioners) awareness, knowledge, and attitude towards innovative products and services by providing scientific and educational information were considered an education-oriented interaction. Interactions which were specifically focused on supporting, funding, or sponsoring research projects were considered research-oriented interactions. Furthermore, the articles included in our review studied various types of outcomes of interactions between pharmaceutical companies and healthcare providers. Four of the ten articles studied how these interactions influenced the prescribing behavior of physicians, for example in the form of increased prescription of brand-name medicines [18, 46, 48, 52]. Four other studies investigated whether interactions between pharmaceutical companies and healthcare providers were associated with ethical dilemmas, such as perceived conflicts of interests [47, 49, 53, 54]. The remaining two studies investigated the effects of bilateral interactions on research output, for example measured through professionals’ scholarly impact [50, 51] (N = 2). Table 4 presents a summary of the results of the studies included in our review.

**Table 4:** Findings in studies.

Type	Outcome	Study	Findings in studies
**Education-oriented interaction**	Prescribing behavior	Taylor, Huecker [18]	Positive association between reported pharmaceutical payments and increased physician-prescribing habits. Small gifts may be as influential as large gifts.
		Yeh, Franklin [52]	Industry payments to physicians are associated with higher rates of prescribing brand-name statins.
	Ethical dilemma	N/A	N/A
	Research output	N/A	N/A
**Research-oriented interaction**	Prescribing behavior	Andersen, Kragstrup [46]	Whereas adherence to international treatment recommendations is not affected by pharmaceutical sponsoring of trials, prescribing behavior is affected.
		Glass [48]	Investigators’ prescribing behavior after the study was not related to relative grant amount. The investigator-pharmaceutical payment relationship in Phase 3 clinical trial is a basic drug development business transaction, with no empirical evidence of ethical compromise.
	Ethical dilemma	Choudhry, Stelfox [47]	Although relationships had no influence on the recommendations, there is a need for appropriate disclosure of financial conflicts of interest for authors of CPGs and a formal process for discussing these conflicts prior to CPG development.
		Fisher and Kalbaugh [54]	Besides financial motivation, US private-sector physicians have a professional identity aligned with an industry-based approach to research ethics. This could facilitate a research enterprise that is characterized by high levels of industry control over research protocols, data analysis, and dissemination of information about new pharmaceuticals.
		Gray [49]	Conflict of norms can result in compromises, self-censorship, and distort independence. A network of social interactions can result in unethical behaviors.
		Henry, Doran [53]	Medical specialists who have research relationships with the pharmaceutical industry are more likely to have multiple additional ties than those who do not have research relationships. Given what is known about reciprocity and the “gift relationship,” each additional tie with industry potentially compounds the relationship and increases the potential for obligation, entanglement, and conflicts of interest.
	Research output	Myers, Shaheen [50]	Pharmaceutical industry sponsorship does not appear to negatively impact response rates to a postal survey.
		Svider, Bobian [51]	Receiving industry contributions greater than $1,000 is associated with greater scholarly impact. In a smaller surgical specialty, direct industry research support—as well as indirect contributions potentially impacts scholarly discourse.

None of the studies included in our review showed interaction between pharmaceutical companies and healthcare organizations but merely between pharmaceutical companies and healthcare professionals. Table 3 presents the investigated population from the included studies.

#### Education-oriented interaction

The two studies in our review which assessed the effect of education-oriented interactions between pharmaceutical companies and healthcare providers both utilized a quantitative, cross-sectional design. The objective of both studies was to determine the association between education sponsored by pharmaceutical companies and the prescribing behavior of physicians. The studies were both conducted in the United States in 2016 and were based on the open payments database which was linked to other secondary data repositories. The open payments database contains information on payments from pharmaceutical companies to healthcare providers and was used in both studies to retrieve information on education-oriented interaction [18, 52]. Both studies conclude that education-oriented interactions between pharmaceutical companies and healthcare providers alter physicians’ prescribing behavior. That is, physicians who engaged in education-oriented interactions, prescribed more brand name drugs [52] and used a specific injection more frequently [18]. Lastly, both studies find a binary effect of education-oriented interactions on physicians’ prescribing behavior. That is, a higher monetary value of the education-oriented interaction does not have a significant influence on physicians’ prescribing behavior.

#### Research-oriented interaction

The eight studies in our review which assessed research-oriented interaction were all conducted in the United States or western European countries and six of the eight studies utilized a cross-sectional research design. The studies furthermore identified three types of effects resulting from research-oriented interaction between pharmaceutical companies and healthcare providers. These are altered prescription behavior, ethical dilemmas, and research-related effects. The studies report that research-oriented interaction can have a negative effect on the practice of physicians [47]. This is for example due to an increase prescription rate of a trial sponsor’s drugs [46]. Similar to education-oriented interactions however, physicians’ prescribing behavior is not influenced by the monetary amount of a research grant [48]. Studies on research-oriented interaction furthermore indicate that physicians with professional identities that are closely aligned with the pharmaceutical company (i.e. physicians who primarily consider themselves entrepreneurs), are more likely to be susceptible to having the pharmaceutical companies assert higher levels of control over various aspects of research projects [54]. Such control can in turn lead to compromises, distort independence, and self-censorship [49].

One study on research-oriented interaction reported that papers published based on joint research projects between pharmaceutical companies and healthcare providers have greater scholarly impact [51]. However, these interactions were not reported to have a methodological influence. That is, one study in the review revealed that survey response rates are not significantly different in pharmaceutical industry-funded research projects compared to university sponsorships [50]. The studies in our review furthermore find no evidence that research-oriented interaction affects physicians’ adherence to international treatment guidelines [46], nor the development of clinical practice guidelines [47]. Lastly, research-oriented interaction was found to have a Matthew-effect. That is, research-oriented interactions between pharmaceutical companies and healthcare providers led to multiple additional relations between the providers and pharmaceutical companies, for example in the form of roles on advisory panels [53].

## Discussion

This study aimed to deepen our understanding of the effects of inter-organizational relations between pharmaceutical companies and healthcare providers, by reviewing the quantitative evidence regarding the effects of bilateral interaction between these organizations. The studies included in our review identified education-oriented and research-oriented interactions as the two main forms of bilateral interactions between pharmaceutical companies and healthcare providers, but vary in terms of the effects under study [18, 46–54]. All studies included in our review report negative or neutral effects of interactions between pharmaceutical companies and healthcare providers. Our findings are thus in line with the literature regarding gifts (i.e. unilateral interactions) from pharmaceutical companies to healthcare providers [55]. However, they do not seem to provide support for the theoretical notion that bilateral interactions between for-profit and not-for-profit organizations have greater value-creating effects [9].

While the type of effects studied for bilateral interactions overlap to a great extent with those studied for unilateral interactions [56, 57, 58], the use of different outcome measures does not explain the deviant findings. Even though we refrained from selecting only those studies which used individual providers as the unit of analysis, all studies in our review focus on effects at the level of individual healthcare professionals [18, 46–54] That is, research predominantly seeks to identify how interactions with pharmaceutical companies affect physician-level outcomes such as prescription behavior, research output, or ethical dilemmas [18, 46, 48, 52]. Outcomes which manifest at an organizational level rather than on the level of individual professionals are overlooked in most studies. While gifts commonly involve specific professionals in an organization, interactions further down the collaboration continuum become more strongly embedded in an organization [9, 40]. Yet, none of the studies in our review focus on such effects, which could provide an explanation for our deviant findings. That is, in case bilateral exchanges of resources only occur at the level of the individual professional, interactions between pharmaceutical companies and healthcare providers might not reach the strategic organizational level. As a result, resource exchanges do not conjoin and the value-creation potential is not fulfilled. Ultimately, the effect of bilateral interactions on more general and overarching health systems outcomes such as costs, quality, and accessibility of care remain unclear.

Secondly, our review revealed that there is a lack of quantitative evidence regarding bilateral interactions between pharmaceutical companies and healthcare providers. Many of the papers which were retrieved by our initial search strategy constituted opinion papers, editorials, or conceptual studies regarding the topic. Few studies hence met the stringent inclusion criteria of our review, even though the topic has been widely discussed in academic journals. The quantitative studies which were ultimately included in our review, predominantly utilized cross-sectional designs and scored relatively low on the quality rating scale (i.e. an average of 3.45). Given the relatively low study quality, there is no definitive answer regarding the desirability of these types of interactions. These considerations could indicate that researchers encounter difficulties, for example feasibility-wise or ethically, to construct high-quality studies regarding bilateral interactions between pharmaceutical companies and healthcare providers. However, given the vast amount of attention paid to this subject and its inherent societal relevance, future research of high empirical quality could make the effects and desirability of bilateral interactions between pharmaceutical companies and healthcare providers clearer.

### Limitations

Our work is subject to some limitations. Given the exploratory nature of our work, we refrained from specifying specific types of interactions or effects in our search strategy. Instead, we focused on generic keywords such as ‘interaction’ or ‘cooperation’ and ‘effect’ or ‘outcome’. As a result, it is possible that some studies were overlooked, which could explain the low number of studies included in the review. However, we refrained from pre-specifying specific types of interactions or effects in order to avoid any a priori bias in our search. Secondly, distinguishing between unilateral and bilateral interactions is a novel approach to the literature regarding interactions between pharmaceutical companies and healthcare providers. As a result, not all types of interactions between pharmaceutical companies and healthcare providers are pre-defined as belonging solely and unambiguously to one of the two categories or were clearly identifiable from the studies. Lastly, we have limited our review to include quantitative studies of bilateral interactions between pharmaceutical companies and healthcare providers. While qualitative studies are able to reveal relevant details of a phenomenon under study, their generalizability is inherently restricted to the empirical setting in which they were studied. Since we aimed to connect two previously unconnected streams of literature, we instead focused exclusively on quantitative studies. These carry a greater degree of external validity, and are hence more generalizable across settings and across theoretical backgrounds. Consequently, qualitative empirical evidence was not included. While a considerable body of literature regarding interactions between pharmaceutical companies and healthcare providers revolves around qualitative research, and we consider this relevant work, we specifically focused on the quantitative effects in order to identify which effects have been empirically tested.

### Further research

Our review identified few empirical studies regarding the effects of bilateral interaction between pharmaceutical companies and healthcare providers. Future research regarding the presence of such interactions and their effects is therefore recommended. Said research should preferably utilize robust designs and be of high methodological quality and study various outcomes at the individual, organizational, and health system level. Furthermore, this study has attempted to reconcile the theoretical framework from the business literature with the empirical research from interaction between pharmaceutical companies and healthcare providers. While both streams of literature study inter-organizational relations between for-profit and not-for-profit organizations, they have remained largely separate. While we have made a first step towards drawing lessons from both research paradigms, we believe that further congruence between these two fields in future research would greatly advance our understanding of this phenomenon on the theoretical as well as the empirical side of this phenomenon. Ultimately, this will enable more adequate identification and explanation of beneficial or adverse effects of interaction between pharmaceutical companies and healthcare providers, and could form the basis for future practical guidelines.

## Conclusion

This study reviewed the empirical literature regarding bilateral interactions between pharmaceutical companies and healthcare providers. Similar to the evidence regarding unilateral interactions, bilateral interactions between pharmaceutical companies and healthcare providers either have no effect or lead to negative outcomes. Bilateral interactions between pharmaceutical companies and healthcare providers hence fail to create the value which theory predicts. However, the existing empirical evidence is limited and largely overlooks outcomes at the organizational or health system level. There is ample opportunity for future research to advance this body of knowledge using robust research designs.

## Supporting information

S1 Table Search strategy PubMed (with MeSH), Cochrane Library and EBSCO. (PDF)

S2 Table Inclusion and exclusion criteria. (PDF)

S3 Table Characteristics of the selected studies. (PDF)

S4 Table Findings in studies. (PDF)

S1 Doc Prisma checklist. (DOC)
