# Supplementary material for: Pharmaceutical companies and healthcare providers: Going beyond the gift – An explorative review
Source: PLoS One. 2018 Feb 7;13(2):e0191856. doi: 10.1371/journal.pone.0191856 (PMC5802853; doi:10.1371/journal.pone.0191856)
Supplement: S1 Table — (PDF) [file pone.0191856.s001.pdf]

Table 1. **Search Strategy PubMed (with MeSH), Cochrane Library and EBSCO.**

Keywords	Synonyms
Pharmaceutical companies	pharmaceutical industr* OR pharmaceutical industry [MeSH] OR pharmaceutical industries [MeSH] OR drug indstr* OR drug industry [MeSH] OR drug industries [MeSH] OR drug compan* OR pharmaceutical compan*)
Healthcare providers	healthcare provider [MeSH] OR healthcare providers [MeSH] OR Health personnel [MeSH] OR physician OR physicians OR general practitioner [MeSH] OR general practitioners [MeSH] OR medical specialist* OR healthcare professional* OR health professional* OR doctor OR doctors OR medical doctor* OR healthcare organization* OR healthcare institution* OR hospital OR hospitals OR healthcare practice* OR healthcare industry [MeSH] OR healthcare industries [MeSH] OR general practice [MeSH]
Interaction	interaction* OR collaboration* OR cooperation* OR public private cooperation [MeSH] OR cooperative* OR collaborative* OR cooperative behavior [MeSH] OR cooperative behaviors [MeSH] OR cooperative behavior* OR cooperative behaviour* OR relation* OR partner* OR public private partnership [MeSH] OR payment* OR grant* OR grants [MeSH] OR sponsor* OR alliance* OR strategic alliance* OR funding* OR contact* OR association* OR connection* OR transaction* OR synerg* OR coalition*
Effect	effect* OR consequence* OR outcome* OR result* OR impact* OR influence* OR conclusion* OR implication*
