# Supplementary material for: Pharmaceutical companies and healthcare providers: Going beyond the gift – An explorative review
Source: PLoS One. 2018 Feb 7;13(2):e0191856. doi: 10.1371/journal.pone.0191856 (PMC5802853; doi:10.1371/journal.pone.0191856)
Supplement: S2 Table — (PDF) [file pone.0191856.s002.pdf]

Table 2. Inclusion and exclusion criteria.

	Inclusion Criteria	Exclusion criteria
**Database**	- Search terms (See Table 1 for the search terms in PubMed and EBSCO)<br>- Year of publication between January 2000 and October 31st, 2016<br>- Language: English<br>- Peer-reviewed	N/A
**Title / Abstract**	The abstract or title of each article had to include a combination of the following keywords: pharmaceutical companies, healthcare providers, interaction, and effects, or variations to the preceding nouns.	- Explicitly mention only unilateral types of interaction or no clear distinction.<br>- Explicitly mention a different aim then investigating an effect of interaction.<br>- Non-empirical study.<br>- Non- quantitative study.<br>- Commentaries, reviews, opinion articles.
**Full-texts**	The article empirically investigates the effect of bilateral interaction between pharmaceutical companies and healthcare providers.	- Non-empirical studies.<br>- Non-quantitative studies.<br>- Studies on unilateral interaction or no clear distinction.<br>- Other stakeholders.<br>- Studies that only described the prevalence.<br>- Studies that describes no interaction.
