# Supplementary material for: Pharmaceutical companies and healthcare providers: Going beyond the gift – An explorative review
Source: PLoS One. 2018 Feb 7;13(2):e0191856. doi: 10.1371/journal.pone.0191856 (PMC5802853; doi:10.1371/journal.pone.0191856)
Supplement: S3 Table — (PDF) [file pone.0191856.s003.pdf]

Table 3. Characteristics of the selected studies.

Study, year	Site	Population (n)	Type of interaction	Type of effect	Study designs	Quality rating score
**Andersen, Kragstrup [46], 2006**	Denmark	Patients treated (5,439 case, 59,574 control)	Research-oriented (sponsoring)	Prescribing behavior	Retrospective cohort study	3
**Choudhry, Stelfox [47], 2002**	N-American and European societies	authors of Clinical Practice Guidelines (CPG) (100)	Research-oriented (CPGs)	Ethical dilemma	Cross-sectional survey	4
**Fisher and Kalbaugh [54], 2012**	United States	Informants (63)	Research-oriented (contract research)	Ethical dilemma	Cross-sectional qualitative study	4
**Glass [48], 2004**	United States	US physicians participating in phase 3 trial (2,108)	Research-oriented (clinical grant)	Prescribing behavior	Cross-sectional quantitative study	4
**Gray [49], 2013**	United States	University medical professor (1)	Research-oriented (funding)	Ethical dilemma	Case study (cross-sectional)	5
**Henry, Doran [53], 2005**	Australia	medical specialists (823)	Research-oriented (sponsoring)	Ethical dilemma	Cross-sectional quantitative study	4
**Myers, Shaheen [50], 2007**	Canada	physicians and nurses (229)	Research-oriented (sponsoring)	Research output	Cross-sectional randomized trial	1
**[51], 2016**	United States	Otolaryngologists (1,515)	Research-oriented (support)	Research output	Quantitative study	4
**Taylor, Huecker [18], 2016**	United States	US ophthalmologists (3011)	Education-oriented	Prescribing behavior	Cross-sectional quantitative study	4
**Yeh, Franklin [52], 2016**	United States	Massachusetts physicians (2444)	Education-oriented	Prescribing behavior	Cross-sectional quantitative study	4
