# Supplementary material for: Pharmaceutical companies and healthcare providers: Going beyond the gift – An explorative review
Source: PLoS One. 2018 Feb 7;13(2):e0191856. doi: 10.1371/journal.pone.0191856 (PMC5802853; doi:10.1371/journal.pone.0191856)
Supplement: S4 Table — (PDF) [file pone.0191856.s004.pdf]

Table 4. Findings in studies.

Type	Outcome	Study	Findings in studies
**Education-oriented interaction**	Prescribing behavior	Taylor, Huecker [18]	Positive association between reported pharmaceutical payments and increased physician-prescribing habits. Small gifts may be as influential as large gifts.
		Yeh, Franklin [52]	Industry payments to physicians are associated with higher rates of prescribing brand-name statins.
	Ethical dilemma	N/A	N/A
	Research output	N/A	N/A
**Research-oriented interaction**	Prescribing behavior	Andersen, Kragstrup [46]	Whereas adherence to international treatment recommendations is not affected by pharmaceutical sponsoring of trials, prescribing behavior is affected.
		Glass [48]	Investigators' prescribing behavior after the study was not related to relative grant amount. The investigator-pharmaceutical payment relationship in Phase 3 clinical trial is a basic drug development business transaction, with no empirical evidence of ethical compromise.
	Ethical dilemma	Choudhry, Stelfox [47]	Although relationships had no influence on the recommendations, there is a need for appropriate disclosure of financial conflicts of interest for authors of CPGs and a formal process for discussing these conflicts prior to CPG development.
		Fisher and Kalbaugh [54]	Besides financial motivation, US private-sector physicians have a professional identity aligned with an industry-based approach to research ethics. This could facilitate a research enterprise that is characterized by high levels of industry control over research protocols, data analysis, and dissemination of information about new pharmaceuticals.
		Gray [49]	Conflict of norms can result in compromises, self-censorship, and distort independence. A network of social interactions can result in unethical behaviors.
		Henry, Doran [53]	Medical specialists who have research relationships with the pharmaceutical industry are more likely to have multiple additional ties than those who do not have research relationships. Given what is known about reciprocity and the "gift relationship," each additional tie with industry potentially compounds the relationship and increases the potential for obligation, entanglement, and conflicts of interest.
	Research output	Myers, Shaheen [50]	Pharmaceutical industry sponsorship does not appear to negatively impact response rates to a postal survey.
		Svider, Bobian [51]	Receiving industry contributions greater than $1,000 is associated with greater scholarly impact. In a smaller surgical specialty, direct industry research support—as well as indirect contributions potentially impacts scholarly discourse.
